# Comparative analysis of ureteroileal anastomotic stricture rates: Bricker versus Wallace techniques in ileal conduit urinary diversion—a single-surgeon study with BMI-matched design and long-term follow-up excluding cancer recurrence bias

**DOI:** 10.3389/fonc.2025.1613772

**Published:** 2025-10-24

**Authors:** Chao Ren, Maolin Xiao, Jie Zhu, Wei Tong, Faxian Yi

**Affiliations:** 1 Department of Urology, Affiliated Hospital of Inner Mongolia Medical University, Hohhot, China; 2 Department of Urology, Affiliated People’s Hospital, Chongqing University, Chongqing, China; 3 Department of Urology, Inner Mongolia Autonomous Region People’s Hospital, Hohhot, China

**Keywords:** bladder cancer, urinary diversion, Bricker technique, Wallace technique, ureteroileal anastomosis, stenosis rate

## Abstract

**Background:**

Ureteroileal anastomotic stricture (UIAS) remains a critical complication following ileal conduit urinary diversion for muscle-invasive bladder cancer (MIBC). Despite widespread use of Bricker and Wallace techniques, comparative outcomes remain debated. This study compares stricture rates between these techniques under standardized surgical conditions, controlling for body habitus and without cancer recurrence-related stenosis.

**Methods:**

A retrospective analysis included 46 patients undergoing laparoscopic ileal conduit diversion by a single surgeon (2017–2021). Patients were stratified into Bricker (n=18) and Wallace (n=28) groups, matched for BMI and comorbidities. Hydronephrosis severity was graded using the Onen system. Statistical analyses utilized Fisher’s exact test, Mann-Whitney U test, and t-test.

**Results:**

No significant difference in hydronephrosis incidence was observed (Bricker: 11.1% vs. Wallace: 0%, p=0.148). However, the Bricker group exhibited longer operative time (301.89 ± 11.76 vs. 281.32 ± 10.15 minutes, p<0.001) and hospitalization duration (18.18 ± 8.22 vs. 11.38 ± 5.11 days, p=0.005). All strictures were asymptomatic (Onen Grade 1).

**Conclusion:**

Both techniques demonstrate comparable safety regarding stricture rates. Wallace anastomosis offers superior operative efficiency, while Bricker requires additional time for technical precision. This study highlights the importance of standardized surgical protocols and long-term surveillance in optimizing urinary diversion outcomes.

## 1 Introduction

Bladder cancer remains the tenth most common malignancy globally, with radical cystectomy and urinary diversion as the cornerstone of treatment for muscle-invasive disease (1). Ureteroileal anastomotic stricture (UIAS), occurring in 1.7–14% of cases, poses significant clinical challenges, including renal impairment and recurrent infections (2–4). The Bricker (separate ureteral implantation) and Wallace (conjoined ureteral anastomosis) techniques dominate clinical practice, yet conflicting evidence persists regarding their comparative efficacy (5–7).

Recent studies report no significant difference in stricture rates between techniques (8, 9); however, heterogeneous cohorts, variable surgical expertise, and confounding factors such as obesity and cancer recurrence limit their validity (10–12). Emerging evidence suggests BMI ≥30 kg/m² independently predicts stricture risk (OR: 3.2) due to technical challenges in ureteral mobilization (13, 14). Furthermore, late-onset hydronephrosis may reflect tumor recurrence rather than anastomotic failure, complicating outcome interpretation (15, 16).

This study addresses these limitations through three methodological innovations: (1) single-surgeon standardization to eliminate operator-dependent variability; (2) BMI-matched cohorts to control adiposity-related confounders; and (3) ≥12-month follow-up. We further extend prior research by analyzing operative efficiency metrics and providing median 42-month follow-up data in the Wallace group, which was particularly critical for Wallace anastomosis where strictures may manifest later (17, 18).

## 2 Methods

### 2.1 Study design and population

A retrospective review included 46 patients undergoing laparoscopic radical cystectomy with ileal conduit diversion by a single surgeon (2017–2021). Inclusion criteria: histologically confirmed MIBC, no prior pelvic radiotherapy, and ≥12 months of follow-up. Written informed consent was obtained from all participants, with approval from the Ethics Committee of Chongqing People’s Hospital.

### 2.2 Surgical techniques

Both techniques used 4–0 polydioxanone (PDS) sutures.

Bricker Anastomosis: Following retrocolic mobilization of the left ureter, the proximal anti-mesenteric border of the ileal conduit was incised longitudinally (~1.5 cm). The left ureter was anastomosed in a tension-free manner with interrupted 4–0 polydioxanone sutures (about 10 knots per ureter), ensuring absence of torsion. A single-J ureteral stent was advanced retrograde into the left renal pelvis prior to anastomosis. The right ureter was anastomosed similarly approximately 5 cm distal to the left anastomotic site. Excess ureteral length was excised to prevent redundancy and kinking.

Wallace Anastomosis: After retrocolic transposition of the left ureter, both ureters were spatulated longitudinally (~2 cm). The medial walls were joined with a continuous 4–0 polydioxanone suture to form a conjoined plate, which was then anastomosed end-to-end to the ileal conduit using continuous sutures. Single-J stents were inserted antegrade midway through the anastomosis.

The choice of technique was based on intraoperative assessment of ureteral length, mobility, and tension, with Bricker preferred when longer mobilization was required.

### 2.3 Hydronephrosis grading

Postoperative hydronephrosis was graded using the Onen system (19).

### 2.4 Statistical analysis

SPSS v16.0 was used for analysis. Fisher’s exact test (categorical variables), Mann-Whitney U test (non-normal distributions), and t-test (normal distributions) were applied. A sensitivity analysis was performed including only patients with ≥12 months of follow-up. Kaplan-Meier analysis for stricture-free survival was attempted but limited by low event rates. Significance was set at p<0.05.

## 3 Results

### 3.1 Baseline characteristics

Groups were balanced for age, sex, BMI, and comorbidities (**Table 1**). Median follow-up was 22.5 months (Bricker) vs. 42.07 months (Wallace, p=0.002).

**Table 1:** Baseline characteristics.

Variable	Bricker (n=18)	Wallace (n=28)	P-value
Age (years)	59.8 ± 7.9	58.5 ± 9.7	0.817
BMI (kg/m²)	24.6 ± 2.9	24.3 ± 3.3	0.890
Male Sex, n (%)	14 (77.8%)	25 (89.3%)	0.522
Hypertension, n (%)	5 (27.8%)	6 (21.4%)	0.658
Diabetes, n (%)	1 (5.6%)	4 (14.3%)	0.635
Neo-adjuvant chemotherapy	2 (1.1%)	0 (1%)	0.148
Follow-up time (mo)	22.51(13.1, 28.4)	42.07(20.3, 54.5)	0.002

### 3.2 Operative and postoperative outcomes

The Bricker group required longer operative time (p<0.001) and hospitalization (p=0.005). Hydronephrosis rates did not differ significantly (11.1% vs. 0%, p=0.148), with all cases classified as Onen Grade 1 (**Table 2**). Sensitivity analysis confirmed no significant change in results when including only patients with ≥12 months of follow-up.

**Table 2:** Operative and postoperative outcomes.

Variable	Bricker	Wallace	P-value
Operative Time (min)	301.9 ± 11.8	281.3 ± 10.2	<0.001
Urine leakage, n (%)	1(5.6%)	4(14.3%)	0.658
Pathological stage			0.595
Tis-T1	4	5	
T2	10	15	
T3	2	5	
T4	2	3	
Hospital Stay (days)	18.2 ± 8.2	11.4 ± 5.1	0.005
Hydronephrosis, n (%)	2 (11.1%)	0 (0%)	0.148

## 4 Discussion

This study provides novel insights into UIAS risk stratification by addressing critical limitations of prior research.

Single-Surgeon Standardization: By eliminating inter-operator variability, our findings isolate technique-specific outcomes. Recent studies emphasize the impact of surgical expertise on anastomotic success (20, 21). Lee et al. (2024) demonstrated that robotic Bricker anastomosis reduced stricture rates by 40% compared to open techniques, highlighting the role of precision in complex ureteral reconstruction (18). Our single-surgeon design ensures uniformity in tissue handling and suture tension—key factors in preventing ischemia-induced fibrosis (22).

BMI-Matched Design: Obesity complicates ureteral mobilization, particularly in Bricker anastomosis requiring retrocolic tunneling (23). Our cohort’s BMI matching (mean 24.5 kg/m²) contrasts with prior studies excluding obese patients (13), enhancing generalizability to real-world MIBC populations. The absence of intergroup BMI differences (p=0.890) mitigated selection bias from historical preferences for lower-BMI candidates in Bricker procedures, a limitation criticized in recent meta-analyses (24). Notably, despite the Bricker group’s longer operative time (301 vs. 281 minutes), no cases of ureteral kinking or ischemia were observed, suggesting meticulous technique can mitigate obesity-related risks.

Exclusion of Cancer Recurrence: There was no cancer recurrence, including at the ureteroileal anastomosis. We report pure technique-related stenosis rates. This distinction is critical, as Kim et al. (2023) found 18% of late hydronephrosis cases were attributable to pelvic recurrence rather than anastomotic failure (25). Our Wallace cohort’s zero stricture rate over a median 42-month follow-up challenges historical concerns about conjoined anastomosis susceptibility to late stenosis (26), possibly due to improved mucosal apposition techniques (27).

Operative Efficiency and Clinical Implications: The Bricker technique’s prolonged operative time (Δ20 minutes) reflects its technical complexity (28). However, this did not translate to higher complications, emphasizing that time invested in precise ureteral alignment may prevent long-term morbidity. Conversely, Wallace anastomosis offers efficiency advantages in complex dissections, particularly when retroperitoneal fibrosis or prior radiation limits ureteral mobility (29).

Emerging Technologies: Indocyanine green (ICG) fluorescence imaging holds promise for further reducing stricture risk by enabling real-time assessment of ureteral perfusion. Smith et al. (2023) reported a 50% reduction in UIAS with ICG use, particularly valuable in Bricker anastomoses where ischemic risk is higher due to retrocolic tunneling (30). Integrating such technologies into standardized protocols could optimize outcomes for both techniques, especially in high-BMI patients or complex dissections.

Limitations and Future Directions: While our study benefits from rigorous design, limitations include its retrospective nature, modest sample size, and disparate follow-up times between groups. The longer follow-up in the Wallace group (median 42 months) strengthens validity for late stricture assessment but may introduce bias; we have therefore toned down claims regarding late-onset strictures. Future multi-center trials should explore the impact of robotic platforms on Bricker/Wallace outcomes (31), biomarkers (e.g., urinary TGF-β1) for early stricture detection (32), and long-term renal function trajectories post-diversion (33).

## 5 Conclusion

Both Bricker and Wallace techniques demonstrate comparable safety profiles when performed meticulously. Wallace anastomosis offers advantages in operative efficiency, particularly relevant for obese patients or complex dissections. Standardization of surgical protocols and extended surveillance remain critical to optimizing outcomes.

## Data Availability

The raw data supporting this study cannot be publicly shared due to institutional data privacy policies and confidentiality agreements. Specific inquiries regarding data points may be directed to the corresponding author under institutional oversight.
