# Expression of concern: comparative analysis of ureteroileal anastomotic stricture rates: Bricker versus Wallace techniques in ileal conduit urinary diversion—a single-surgeon study with BMI-matched design and long-term follow-up excluding cancer recurrence bias

**DOI:** 10.3389/fonc.2026.1912670

**Published:** 2026-06-30

**Authors:** Frontiers Editorial Office

With this notice, Frontiers states its awareness of concerns regarding reference reliability of “Comparative analysis of ureteroileal anastomotic stricture rates: Bricker versus Wallace techniques in ileal conduit urinary diversion—a single-surgeon study with BMI-matched design and long-term follow-up excluding cancer recurrence bias” published on 24th October 2025. An investigation is currently being conducted by the Frontiers Research Integrity team, in accordance with COPE guidelines and Frontiers’ applicable policies and procedures. This expression of concern has been posted while Frontiers awaits the outcome of that investigation. It will be updated accordingly after that time.

